# Core principles for infection prevention in hemodialysis centers during the COVID-19 pandemic

**DOI:** 10.1017/ice.2020.109

**Published:** 2020-04-06

**Authors:** Gang Chen, Yangzhong Zhou, Lei Zhang, Ying Wang, Rong-rong Hu, Xue Zhao, Dan Song, Jing-hua Xia, Yan Qin, Li-meng Chen, Xue-mei Li

**Affiliations:** 1 Nephrology Department, Peking Union Medical College Hospital, Peking Union Medical College, Chinese Academy of Medical Sciences, Peking, China; 2 Internal Medicine, Peking Union Medical College Hospital, Peking Union Medical College, Chinese Academy of Medical Sciences, Peking, China

*To the Editor—*The COVID-19 outbreak began at the end of December 2019, and >600,000 confirmed cases had been reported worldwide by the end of March 2020. The World Health Organization declared a global pandemic.^1^ Nosocomial transmission was severe in some locations, and the burden to the health system was extreme.^2^ Hemodialysis centers, which generally serve high volumes of highly mobile dialysis patients, have an exceptionally high risk of exposure during this outbreak period. In a general tertiary-care hospital, dialysis centers routinely accept patients from outpatient clinics and emergency rooms, further adding to the difficulty of preventing nosocomial infection.^3^ Dialysis patients, commonly regarded as immune compromised, are likely to develop severe illness as a result of close contact in a medical unit.

Droplet spread and close contact are the main routes of COVID-19 transmission.^4^ Thus, the hemodialysis center in our hospital implemented multiple strategies for infection prevention, including area management and integrated symptom monitoring, in the context of this pandemic.

Based on various levels of exposure to the mobile population, our hospital environments were classified as low-risk, medium-risk, high-risk, and extremely high-risk, and the dialysis center belongs to the high-risk category. We avoid moving across the area by designing a specific walking route for our patients entering the hemodialysis center. Medical staff wears personal protective equipment (PPE) when inter-area contact is inevitable. For example, N95 masks and protective glasses are required when entering the fever clinic. We advise the use of hand sanitizer whenever staff return to the hemodialysis center. In the dialysis center, a 1-way route is followed by our patients, and mask-wearing and hand sanitizing by the patients are ensured. During the dialysis session, we provide necessary education on maintaining social distancing and self-protection. Between the 2 dialysis shifts, we strictly leave at least 30 minutes for environmental and air disinfection, and we utilize a chlorine-containing disinfectant to clean our dialysis facilities.^6–8^

We monitor and respond to our regular patients’ symptoms in an integrated way. Between the dialysis sessions, we strictly record the body temperatures and any suspicious respiratory symptoms of our patients. For patients referred from other departments in the hospital, we collect records of their contact history, temperature, and potential warning symptoms before admission. Based on this information, all of our patients are classified into 3 categories (Table 1). A negative SARS-CoV-2 swab test is needed for patients in category C before their dialysis session can be scheduled. In emergency cases, we perform continuous renal replacement therapy (CRRT) in a separate place, preferably in a negative-pressure ward, before completely ruling out COVID-19 for these patients.^5,6^ Notably, patients with a positive swab test are sent to designated hospitals for further treatment.

**Table 1.** High-Risk and Suspicious Patient Identification and Classification Management

Category	Definition	Management
A: Temperature warning	Temperature 37.0~37.2°C in the past 14 d, without other symptoms	Close observation
B: Symptom warning	Suspicious symptoms (ie, sore throat, cough, and diarrhea, etc) in the past 14 d; but the temperature within the normal range	Separate dialysis and close observation
C: High-risk warning	Temperature >37.3°C within 14 d, together with suspicious history, or respiratory symptoms, or chest imaging abnormalities	Screen SARS-CoV-2 swab; send positive patients to the specific hospitals; arrange negative patients for separate dialysis and close observation

Medical staff are strictly required to maintain hand hygiene and to wear a mask at work. N95 masks and protective goggles are used when operating CRRT for patients in category C. The equipment used is disinfected between patients, and medical waste is packed and labeled separately to avoid potential contamination.^7^ The waste liquid generated during CRRT is discharged according to the requirements of the medical wastewater discharge standards.^8^

In addition to the strategies summarized above, we promote work–life balance for staff and encourage patients to take the initiative to participate. Our hemodialysis center has strived to achieve zero infection during the ongoing COVID-19 outbreak.
